# Worse Outcome in Stroke Patients Treated with rt-PA Without Early Reperfusion: Associated Factors

**DOI:** 10.1007/s12975-017-0584-9

**Published:** 2017-11-07

**Authors:** Ramón Iglesias-Rey, Manuel Rodríguez-Yáñez, Emilio Rodríguez-Castro, José Manuel Pumar, Susana Arias, María Santamaría, Iria López-Dequidt, Pablo Hervella, Clara Correa-Paz, Tomás Sobrino, Denis Vivien, Francisco Campos, Mar Castellanos, José Castillo

**Affiliations:** 1 Clinical Neurosciences Research Laboratory, Department of Neurology, Clinical University Hospital, Universidade de Santiago de Compostela, Health Research Institute of Santiago de Compostela (IDIS), 15706 Santiago de Compostela, Spain; 2 Department of Neuroradiology, Clinical University Hospital, Universidade de Santiago de Compostela, Health Research Institute of Santiago de Compostela (IDIS), 15706 Santiago de Compostela, Spain; 3 Inserm, Inserm, UMR-S U1237, Physiopathology and Imaging of Neurological diseases, GIP Cyceron, Caen Normandie University, 14073 Caen, France; 4 CHU de Caen, Department of Clinical Research, Caen University Hospital, 14000 Caen, France; 5 Department of Neurology, Biomedical Research Institute, University Hospital A Coruña, 15006 Corunna, Spain

**Keywords:** Blood-brain barrier, Critical care, Hemorrhage transformation, Ischemic stroke, Prognosis

## Abstract

Based on preclinical studies suggesting that recombinant tissue plasminogen activator (rt-PA) may promote ischemic brain injuries, we investigated in patients the possible risk of worse clinical outcome after rt-PA treatment as a result of its inability to resolve cerebral ischemia. Here, we designed a cohort study using a retrospective analysis of patients who received treatment with intravenous (4.5-h window) or intraarterial rt-PA, without or with thrombectomy. Controls were consecutive patients who did not receive recanalization treatment, who met all inclusion criteria. As a marker of reperfusion, we defined the variable of early neurological improvement as the difference between the score of the National Institute of Health Stroke Scale (NIHSS) (at admission and 24 h). The main variable was worsening of the patient’s functional situation in the first 3 months. To compare quantitative variables, we used Student’s *t* test or the Mann-Whitney test. To estimate the odds ratios of each independent variable in the patient’s worsening in the first 3 months, we used a logistic regression model. We included 1154 patients; 577 received rt-PA, and 577 served as controls. In the group of patients treated with rt-PA, 39.4% who did not present clinical reperfusion data developed worsening within 3 months after stroke compared with 3.5% of patients with reperfusion (*P* < 0.0001). These differences were not significant in the control group. In summary, administration of rt-PA intravenously or intraarterially without reperfusion within the first 24 h may be associated with a higher risk of functional deterioration in the first 3 months.

## Introduction

Stroke is the second leading cause of death in developed countries, the second leading cause of dementia, and the leading cause of major disability in adults, with an increasing incidence because of the progressive aging of the population in such countries. Since 1995, despite its complications, recombinant tissue plasminogen activator (rt-PA) administered intravenously alone or subsequently in combination with intraarterial administration or with mechanical thrombectomy is the only drug treatment for acute ischemic stroke [1–7]. Besides the unquestionable benefit from its thrombolytic activity, consistent evidence has accumulated on the neurotoxic effect of rt-PA both in vitro and in vivo, including in experimental models of cerebral ischemia [8–14]. Mechanisms by which rt-PA causes these neurotoxic effects have not been fully elucidated, mainly due to their multifactorial and time-dependent activity.

The clinical evidence on the neurotoxicity associated with rt-PA treatment following ischemic stroke is still debated, and it is of crucial importance to reduce risks after administration of the thrombolytic agent for a better patient management [15, 16]. In the present work, we thus decided to set-up a clinical study based on the hypothesis that rt-PA could be a “Janus” drug. Our hypothesis was based on two possible scenarios: (1) If the thrombolytic activity of rt-PA is effective and cerebral ischemia rapidly resolved, the blood-brain barrier remains intact with the rt-PA maintained within the vascular compartment, thus leading to a better clinical outcome; (2) if the rt-PA cannot play its thrombolytic action thus leading to a prolonged cerebral ischemia, rt-PA could then enhance damages of the blood-brain barrier and of the cerebral parenchyma, thus leading to a worse clinical outcome [17].

## Materials and Methods

### Study Design

A retrospective cohort study (*n* = 1154) was designed using a prospective registry of acute ischemic stroke patients (BICHUS) approved by the Ethics Committee of Galicia.

Between January 2008 and October 2016, 577 patients who were treated with intravenous (with ECASS II criteria [18] modified with a therapeutic window ≤ 4.5 h and with no age limit) or intraarterial rt-PA, without or with thrombectomy (who previously received rt-PA), were analyzed to be included in the study. On the other hand, 577 controls were defined as ischemic stroke patients not treated with rt-PA, who were selected after each case included. Both groups met all the inclusion criteria and none of the exclusion criteria.

### Inclusion and Exclusion Criteria

The inclusion criteria were as follows: (1) Ischemic stroke patients attended by a neurologist according to common protocol [19] and admitted to the stroke unit, (2) fewer than 6 h of evolution (no wake-up strokes were included), (3) neuroimaging on admission, (4) previous modified Rankin Scale (mRS) < 2, and (5) without previous stroke and without lacunar syndrome (LACI).

The exclusion criteria were as follows: (1) institutionalized patients, (2) comorbidity and life expectancy < 1 year, (3) without subsequent diagnostic confirmation, (4) lacunar infarctions, and (5) loss of follow-up at 3 months were excluded.

### Clinical Variables

The clinical variables analyzed were age, sex, axillary temperature at admission, history of arterial hypertension (at least two blood pressure measurements greater than 140/85 mmHg or with antihypertensive treatment), diabetes (previous diagnosis or with antidiabetic treatment), alcoholism (> 300 g of alcohol per week), smoking (habitual smoker or until the last year), dyslipidemia (at least a previous determination of total cholesterol > 230 mg/dL or antihyperlipidemic treatment), peripheral arterial disease, coronary disease, atrial fibrillation, known carotid disease, and prior transient ischemic attack. Classification according to Oxfordshire Community Stroke Project (OSCP) criteria [20], National Institute of Health Stroke Scale (NIHSS) at entry and at 24 h, start-inclusion time and start-needle time, TOAST classification [21], hemorrhage transformation (according to ECASS II criteria [18]), and mRS at discharge and at 3 months. An accredited neurologist rated the scales. For this study, blood glucose, leukocytes, fibrinogen, C-reactive protein, erythrocyte sedimentation rate, and albumin per gram of creatinine were collected at the time of admission. A second neuroimaging study was performed on all patients between the fourth and seventh day after admission or immediately if neurological impairment was detected (defined as the ≥ 4-point increase in NIHSS). During their hospitalization and after hospital discharge, the patients were attended by physicians and physiotherapists of the Rehabilitation Service of the Clinical University Hospital of Santiago de Compostela.

### Main Outcomes

According to previous studies [22–24], as a marker of reperfusion, we defined the variable of early neurological improvement as the difference between the score of the NIHSS determined at admission and at 24 h (an improvement of 8 points in NIHSS). The study’s main variable was the worsening of the patient’s functional situation in the first 3 months. This variable was determined as the difference of the mRS between hospital discharge and 3 months ± 15 days (mRS from discharge to 3 months). We defined the worsening as a value mRS from discharge to 3 months < 0. Death at any time since inclusion of the patient was classified as worsening. An mRS at 3 months ≤ 2 was defined as a good outcome.

### Statistical Analyses

Results were expressed as percentages for categorical variables and as mean (standard deviation [S.D.]) or median and range (25th and 75th percentiles) for the continuous variables, depending on whether their distribution was normal. The Kolmogorov-Smirnov test was used for testing the normality of the distribution. Proportions were compared using the chi-square or Fisher test, while continuous variables between groups were compared with Student’s *t* or the Mann-Whitney tests, depending on whether their distribution was normal. Bivariate correlations were performed using Pearson’s (normally distributed variables) or Spearman (variables without normal distribution) coefficients.

The association of fibrinolytic treatment, with and without reperfusion, on worsening functional outcome (mRS from discharge to 3 months < 0) was assessed by logistic regression analysis models. Each logistic regression analysis model was adjusted for the independent variables in the bivariate analysis. Results were expressed as adjusted odds ratios (ORs) with the corresponding 95% confidence intervals (95% CI). On the other hand, receiver operating characteristic (ROC) curve analysis was used to compare the early neurological improvement and the improvement of the mRS from discharge at 3 months ± 15 days, as a clinical marker of an effective reperfusion.

A *P* value < 0.05 was considered to be statistically significant in all tests. The statistical analysis was conducted in SPSS 21.0 (IBM, Chicago, IL, USA) for Mac.

## Results

### Longitudinal Studies of Groups

Seven-hundred three patients received fibrinolytic therapy; 499 were treated with intravenous rt-PA, 99 with intraarterial rt-PA, (with or without thrombectomy), and 105 with intravenous rt-PA plus intraarterial rt-PA (with or without thrombectomy). We excluded 126 individuals from this study: 59 were institutionalized, 44 due to lack of follow-up, 2 due to comorbidities, and 21 due to subsequent diagnostic confirmation of lacunar infarction. Therefore, 1154 patients were included in this study, 577 of whom received rt-PA (387 intravenous, 93 intraarterial, and 97 intravenous plus intraarterial with or without thrombectomy), and 577 controls.

Comparison between the rt-PA-treated group and the control group is shown in Table 1. In summary, patients receiving fibrinolytic therapy were worse neurologically on admission and were included in less time. In patients treated with rt-PA, the onset-needle time was 224.1 ± 39.6 min, and the percentage of hemorrhagic transformation was higher. The mRS at 3 months was better, as was the percentage of patients with a good outcome.

**Table 1:** Bivariate analysis between patients treated (fibrinolytic therapy group) and not treated (control group) with rt-PA

	Fibrinolytic therapy *n* = 577	Control *n* = 577	*P*
Age, years	71.7 ± 12.2	70.7 ± 13.4	0.062
Men, %	50.4	55.3	0.111
Previous mRS	0 [0, 1]	0 [0, 1]	0.054
History of high blood pressure, %	62.4	60.7	0.586
History of diabetes, %	19.4	23.7	0.086
History of alcoholism, %	8.7	11.3	0.169
History of smoking, %	13.9	15.6	0.455
History of dyslipidemia, %	40.2	40.4	1.000
Peripheral arterial disease, %	7.1	5.7	0.400
Ischemic heart disease, %	11.8	11.4	0.927
Atrial fibrillation, %	21.3	22.7	0.619
Known carotid disease, %	1.2	0.9	0.773
Previous TIAs, %	1.9	0.9	0.207
OSCP			< 0.0001
TACI, %	45.1	28.8	
PACI, %	48.5	49.4	
POCI, %	6.4	21.8	
NIHSS at admission	14 [10, 18]	9 [6, 16]	< 0.0001
NIHSS at 24 h	8 [5, 16]	8 [6, 15]	0.237
≥ 8 points of the NIHSS in the first 24 h, %	24.8	2.6	< 0.0001
Start-inclusion time, min	175.6 ± 40.6	239.9 ± 69.1	< 0.0001
Start-needle time, min	224.1 ± 39.6	–	
TOAST			0.458
Large vessel disease, %	33.4	30.7	
Cardioembolic, %	39.2	39.0	
Indeterminate, %	27.4	30.3	
Hemorrhagic transformation			< 0.0001
Asymptomatic, %	5.7	1.4	
Symptomatic, %	1.9	0.9	
mRS at discharge	4 [2, 5]	4 [2, 5]	0.964
mRS at 3 months	2 [1, 4]	4 [3, 6]	< 0.0001
Good outcome at 3 months, %	53.8	24.1	< 0.0001
Axillary temperature at admission, °C (*n* = 1154)	36.3 ± 0.6	36.3 ± 0.5	0.926
Blood glucose at admission, mg/dL (*n* = 1154)	135.7 ± 50.4	139.9 ± 61.5	0.199
Leukocytes at admission, ×10^3^/mmc (*n* = 1154)	9.1 ± 3.4	9.1 ± 3.1	0.926
Fibrinogen at admission, mg/dL (*n* = 926)	416.6 ± 116.1	425.9 ± 88.8	0.176
C-reactive protein at admission, mg/dL (*n* = 894)	3.9 ± 4.8	3.6 ± 3.6	0.249
Sedimentation rate at admission, mm/h (*n* = 999)	22.2 ± 22.4	24.1 ± 21.2	0.187
Albuminuria, mg/g of creatinine (*n* = 785)	7.1 ± 33.2	4.4 ± 26.3	0.199

**Fig. 1:**
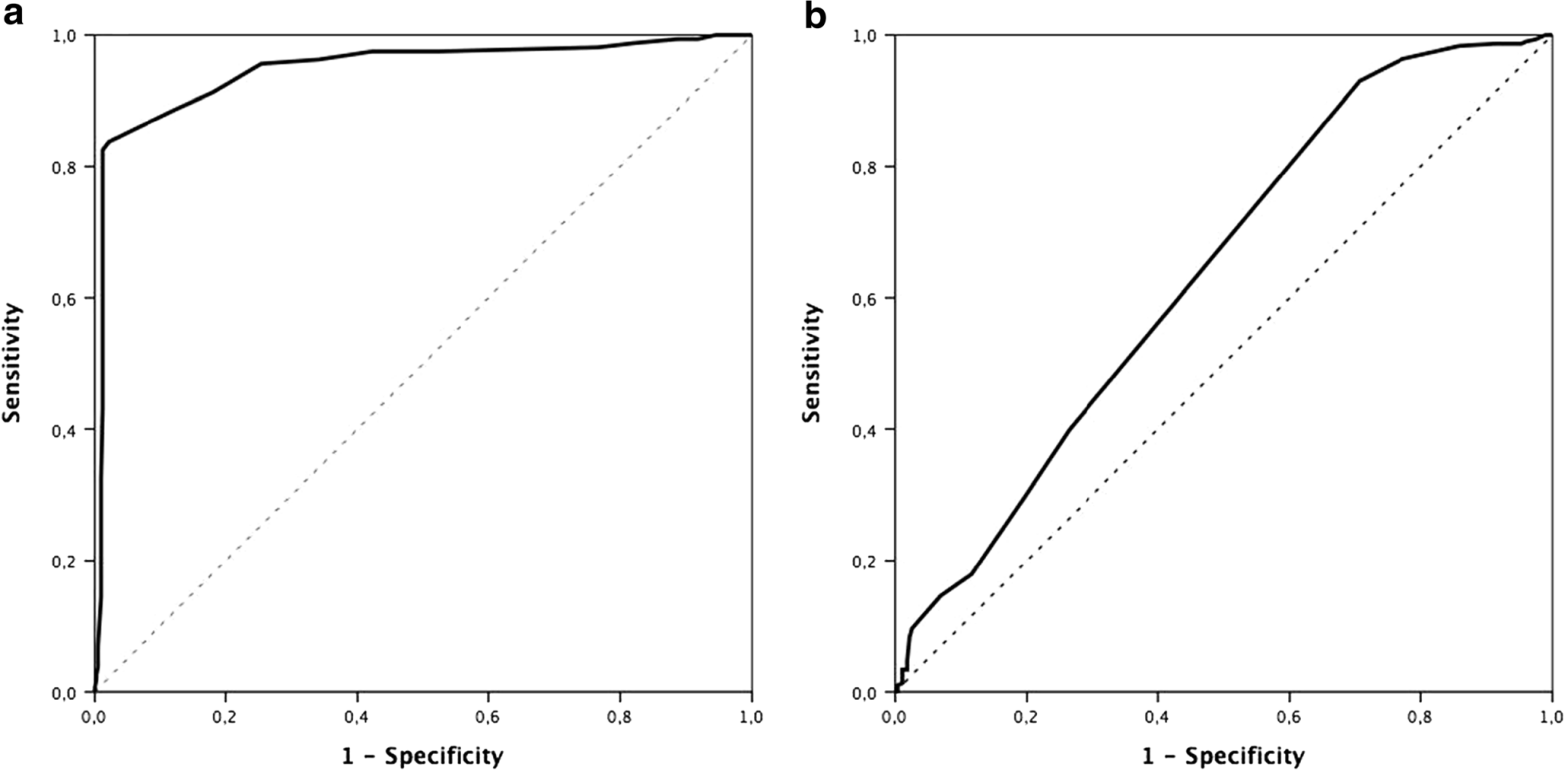
ROC curve. Main variable—improvement of mRS from discharge to 3 months. Influence of the difference among the NIHSS in the first 24 h and the main variable. **a** Patients with fibrinolytic treatment. For a cutoff point of 8, the sensitivity is 82.5% and the specificity is 98.8%. Area under the curve 0.948 (CI 95% 0.924–0.971, *P <* 0.0001). **b** Patients without fibrinolytic treatment. Area under the curve 0.636 (CI 95% 0.591–0.682, *P* = 0.023). It is not possible to establish a cutoff point

In our series, in patients treated with rt-PA, an improvement of 8 points in NIHSS in the first 24 h was identified as a clinical marker of effective reperfusion, with a sensitivity of 82.5% and a specificity of 98.8% (area under the curve 0.948; CI 95% 0.924–0.971, *P* < 0.0001). It was not possible to establish the cutoff point in the control group (Fig. 1). The percentage of patients with clinical reperfusion marker was higher in patients treated with rt-PA (24.8 vs. 2.6%, *P* < 0.0001). This reperfusion was 12.1% in patients treated intravenously, 65.6% intraarterially, and 51.5% intravenously and intraarterially combined (*P* < 0.0001).

In the first 3 months of evolution, 240 patients (20.8%) presented worsened functioning. The percentage of worsening in the reperfused patients was similar in the three therapeutic groups (4.1, 3.2, and 3.3%, respectively, *P* = 0.402). The bivariate study (Table 2) among patients who worsened and those who improved (*n* = 461, 39.2%) or remained stable (*n* = 453, 39.9%) showed differences in the percentage of alcoholism, smoking, clinical reperfusion, and treatment with rt-PA. The time between onset-inclusion and onset-needle was greater in patients who worsened. Although mRS at discharge was better in the group of patients who worsened, mRS and the good prognosis at 3 months was significantly worse.

**Table 2:** Bivariate analysis between the group of patients with and without functional outcome worsening (mRS from discharge to 3 months < 0)

	No *n* = 914	Yes *n* = 240	*P*
Age, years	70.9 ± 12.1	72.3 ± 11.8	0.131
Men, %	53.5	50.4	0.424
Previous mRS	0 [0, 1]	0 [0, 1]	0.307
History of high blood pressure, %	62.6	57.5	0.157
History of diabetes, %	21.0	23.8	0.378
History of alcoholism, %	9.0	13.8	0.039
History of smoking, %	16.2	9.2	0.006
History of dyslipidemia, %	39.1	45.0	0.104
Peripheral arterial disease, %	6.2	7.1	0.657
Ischemic heart disease, %	11.1	13.8	0.258
Atrial fibrillation, %	23.2	17.5	0.066
Known carotid disease, %	1.2	0.4	0.478
Previous TIAs, %	1.5	0.8	0.546
OSCP			0.094
TACI, %	37.3	35.4	
PACI, %	47.2	55.8	
POCI, %	15.5	8.8	
NIHSS at admission	12 [8, 17]	11 [7, 16]	0.062
NIHSS at 24 h	8 [5, 15]	10 [6, 16]	0.071
≥ 8 points of NIHSS in the first 24 h, %	16.5	2.9	< 0.0001
Start-inclusion time, min	198.7 ± 57.9	210.1 ± 66.7	0.016
Start-needle time, min (*n* = 577)	221.5 ± 39.6	230.2 ± 39.3	0.017
TOAST			0.535
Large vessel disease, %	31.3	35.0	
Cardioembolic, %	39.4	37.9	
Indeterminate, %	29.3	27.1	
rt-PA treatment, % (*n* = 577)	43.9	73.3	< 0.0001
Hemorrhagic transformation			0.899
Asymptomatic, %	3.6	3.3	
Symptomatic, %	1.3	1.7	
mRS at discharge	4 [2, 5]	3 [2, 4]	< 0.0001
mRS at 3 months	3 [1, 5]	4 [3, 5]	< 0.0001
Good outcome at 3 months, %	40.6	18.3	< 0.0001
Axillary temperature at admission, °C (*n* = 1154)	36.3 ± 0.6	36.4 ± 0.6	0.206
Blood glucose at admission, mg/dL (*n* = 1154)	136.8 ± 54.9	141.8 ± 61.0	0.217
Leukocytes at admission, ×10^3^/mmc (*n* = 1154)	8.9 ± 3.2	9.2 ± 3.2	0.360
Fibrinogen at admission, mg/dL (*n* = 926)	420.5 ± 100.1	422.2 ± 119.7	0.831
C-reactive protein at admission, mg/dL (*n* = 894)	3.7 ± 4.2	4.1 ± 4.9	0.227
Sedimentation rate at admission, mm/h (*n* = 999)	22.8 ± 21.3	24.0 ± 23.9	0.464
Albuminuria, mg/g of creatinine(*n* = 785)	5.6 ± 30.0	6.7 ± 30.7	0.680

### Treatment with rt-PA and Relationship to Worse Clinical Outcome

**Fig. 2:**
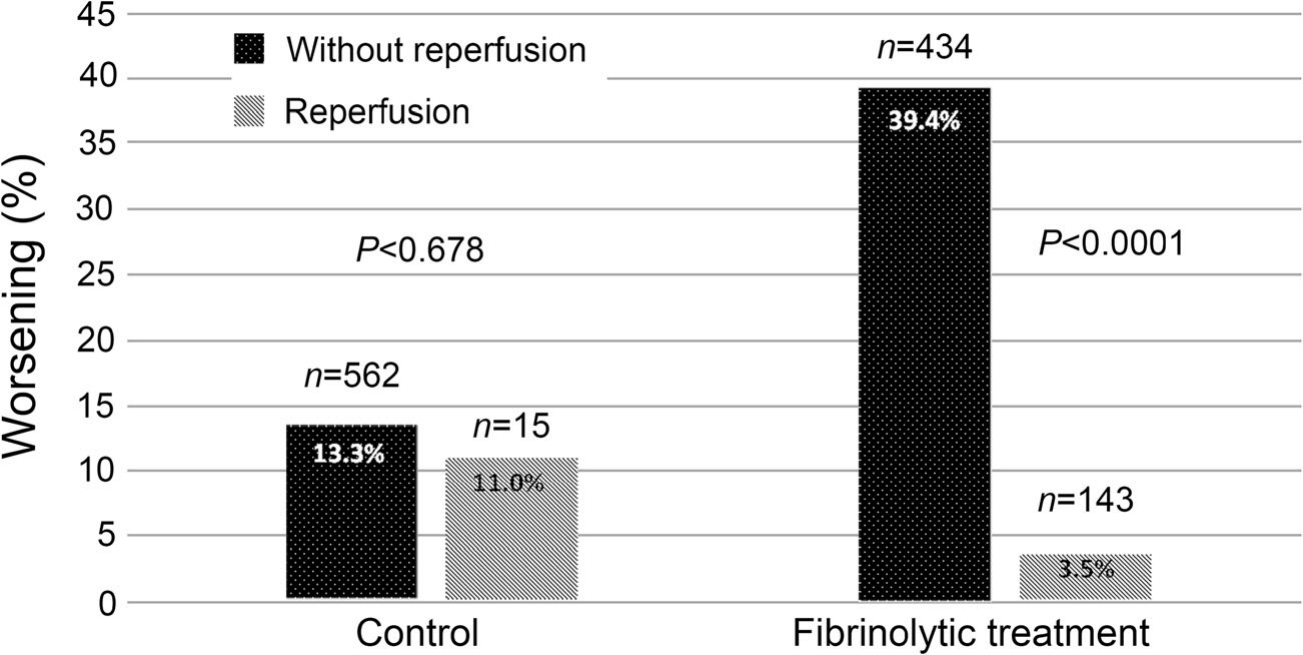
Percentage of worsening in the first 3 months in the group of patients treated with rt-PA and in the control compared to the existence of clinical criteria of reperfusion

In the group of patients treated with rt-PA, 39.4% who did not present clinical reperfusion data developed worsening within 3 months after stroke compared with 3.5% of patients with reperfusion (*P* < 0.0001). These differences were not significant in the control group (Fig. 2).

In the logistic repression model (Table 3), reperfusion was associated with a significant improvement in clinical outcome (OR 0.08, CI 95% 0.04–0.17, *P* < 0.0001), whereas fibrinolytic treatment with rt-PA is a factor independently associated with clinical worsening (OR 4.98, CI 95% 3.60–6.89, *P* < 0.0001). However, rt-PA treatment associated with reperfusion is the independent factor most strongly associated with patient improvement (OR 0.06, CI 95% 0.02–0.14, *P* < 0.0001).

**Table 3:** Crude and adjusted OR of functional outcome worsening (mRS from discharge to 3 months < 0) for fibrinolytic treatment with or without reperfusion

Independent variables	OR*	CI 95%	*P*	OR**	CI 95%	*P*
History of alcoholism	1.62	1.05–2.49	0.029	1.13	0.59–2.14	0.710
History of smoking	0.61	0.19–0.98	0.007	0.53	0.26–1.06	0.074
Start-inclusion time	0.96	0.89–0.99	0.016	0.99	0.56–1.83	0.957
Start-needle time	0.85	0.43–0.97	0.018	0.96	0.55–1.77	0.963
Reperfusion	0.15	0.07–0.33	< 0.0001	0.08	0.04–0.17	< 0.0001
Fibrinolytic treatment	3.52	2.57–4.82	< 0.0001	4.98	3.60–6.89	< 0.0001
Fibrinolytic treatment by reperfusion				0.06	0.02–0.14	< 0.0001

*Unadjusted logistic regression model

**Adjusted logistic regression model

The computed tomography study performed at the 4th–7th days reflected a similar lesion volume in patients with and without fibrinolytic treatment (55.2 ± 80.2 cm^3^ and 56.5 ± 87.8 cm^3^, *P* = 0.851). We found higher lesion volume in patients with worse clinical outcome at 3 months, although the difference did not reach significance (66.7 ± 87.1 cm^3^ vs. 51.6 ± 80.1 cm^3^, *P* = 0.051). If we included the lesion size in the logistic regression model (Table 3), results do not vary (reperfusion (OR 0.07, CI 95% 0.03–0.24, *P* < 0.0001); fibrinolytic treatment (OR 4.19, CI 95% 3.12–7.13, *P* < 0.0001). In the same line, we determined that anticoagulant or antiplatelet therapy were factors independently associated with clinical prognosis at 3 months in the group with functional outcome worsening (49.3% with anticoagulant and 50.7% with antiplatelet, *P* = 0.119).

Only in the group of patients treated with rt-PA, some biological markers associated with inflammation (temperature, leukocytes, C-reactive protein, and erythrocyte sedimentation rate) were significantly higher in patients who did not present clinical reperfusion data (Table 4).

**Table 4:** Biological signatures of ischemic stroke patients treated with rt-PA by reperfusion groups

	No reperfusion *n* = 434	Reperfusion *n* = 143	*P*
Axillary temperature at admission, °C (*n* = 577)	36.4 ± 0.6	36.0 ± 0	< 0.0001
Blood glucose at admission, mg/dL (*n* = 577)	140.2 ± 54.5	122.0 ± 31.4	< 0.0001
Leukocytes at admission, ×10^3^/mmc (*n* = 577)	9.4 ± 3.5	7.8 ± 2.7	< 0.0001
Fibrinogen at admission, mg/dL (*n* = 505)	422.1 ± 118.2	398.5 ± 107.3	0.053
C-reactive protein at admission, mg/dL (*n* = 545)	4.4 ± 5.0	2.3 ± 3.6	< 0.0001
Sedimentation rate at admission, mm/h (*n* = 565)	24.7 ± 23.4	14.7 ± 16.9	< 0.0001
Albuminuria, mg/g of creatinine (*n* = 415)	7.4 ± 33.2	6.4 ± 33.3	0.795

## Discussion

rt-PA is still the gold standard treatment for acute ischemic stroke; however, this is not an innocuous treatment. Although in the clinical practice it has been possible to minimize the risk of hemorrhagic complication, the use of rt-PA in non-reperfused patients is associated with a worse prognosis within 3 months of having an ischemic stroke after ischemic onset.

Therefore, despite the long experience using this treatment, its use should not be considered trivial, and other risks besides hemorrhage must be considered after use. The results in this study confirm that this treatment’s benefits happen only when the occluded artery is successfully reperfused. Thus, the absence of response to intravenous rt-PA should force clinicians to start rescue therapies.

After administration in the circulation, rt-PA acts first as an endogenous thrombolytic enzyme; however, then if this enzyme diffuses in the cerebral parenchyma, by crossing the healthy blood-brain barrier and to a higher extend the damaged blood-brain barrier [25], tPA may be able to originate mechanisms involving NMDA receptors signaling [8], ranging from an increase in synaptic plasticity to neurotoxicity, through pathways dependent or independent of tissue plasminogen [10, 11, 17].

Based on preclinical studies, it has been proven that rt-PA may promote neurotoxicity through its action on extrasynaptic GluN2D-containing NMDARs, whereas it could have neuroprotective effects by activating synaptic GluN2A-containing NMDARs [10, 26, 27]. Despite this possible neuroprotective effect, exogenous rt-PA is the paradigm of in vitro or in vivo excitotoxicity mediated by overactivation of NMDARs [10, 14, 17].

rt-PA is secreted as a single-chain (sc) form, which in presence of plasmin is converted in a two-chain (tc) form by cleavage of Arg275-Ile276 region. Both forms have the same fibrinolytic activity, but they differ in the activation of the N-methyl-D-aspartate receptor (NMDAR). The sc-rt-PA form activates the NMDAR, leading to calcium entry and excitotoxic neuronal death, whereas the tc-rt-PA form inhibits NMDAR and its secondary neurotoxicity [11, 28]. In animal models, the rt-PA-mediated neurotoxicity varies in relation to the ratio between the sc and tc forms [29], data recently confirmed in human [28]. Interestingly, patients treated with rt-PA have been reported to develop more seizures than patients who are not. This potential pro-epileptic effect found in patients is in agreement with the reported capacity of rt-PA (especially its sc form) to promote NMDA receptor signaling.

Experimental and clinical evidence has shown an association between the administration of rt-PA with disruption of the blood-brain barrier and with the consequent risk of edema and hemorrhagic transformation [11, 16, 30], mainly through the overexpression of metalloproteinases [11, 31, 32]. However, after the clinical experience acquired with the use of rt-PA, these complications are not too common [33, 34].

Following a stroke, neurophysiological processes associated with recovery often begin very early after the onset of stroke (from hours to days) and may plateau in months, depending on the specific neurologic deficit [35]. Our present study confirms our initial hypothesis: in patients treated with rt-PA who do not reperfuse in the first 24 h, the probability of a functional worsening is almost five-fold, independent of an increased risk of hemorrhagic transformation. Therefore, we contemplate that the possible rt-PA-induced toxicity in acute stroke may be the responsible of increase brain damage or delaying recovery mechanisms after the onset. We consider that the inclusion of patients treated with rt-PA from a university hospital that has a stroke unit and a protocolized management gives value and consistency to these conclusions. Although this worsening is likely to be associated with NMDA receptor-mediated neurotoxicity, this study’s design—clinical and observational—does not allow confirmation of the molecular mechanism-driven worse outcome. Association of worsening with the presence of markers of inflammation means that the inflammatory environment hinders reperfusion, or that in itself conditions the worse evolution that these patients present. It is thus interesting to note that tPA was also reported to promote transmigration of inflammatory cells across the blood-brain barrier, an effect dependent of NMDA receptors expressed on endothelial cells [36].

Modification of the NIHSS score within 24 h after the administration of a thrombolytic agent has been shown to be a valid criterion for estimating cerebral reperfusion and with a good relation to recanalization as demonstrated by angiography or ultrasound. However, in the absence of angiographic or ultrasonographic confirmation, the clinical criteria to define effective reperfusion are both unanimous and possibly vary in relation to the sample studied. An improvement of ≥ 10 points or ≥ 20% between baseline and at 24 h has nevertheless shown a good relationship [37–40]. Here, we used the cutoff point ≥ 8 because the sensitivity and specificity were higher (with a cutoff point ≥ 10, the sensitivity was 84.3%, but the specificity was 86.1%).

Despite the efficacy and safety of fibrinolytic treatment in lacunar infarcts [41, 42], we decided to exclude these patients from the study for several reasons: (1) in an unknown proportion of lacunar infarcts, the pathologic basis is lipohyalinosis, which should not be a subsidiary of the administration of rt-PA [42]. (2) Markers of fibrinolysis, coagulation, endothelial, and inflammation are different [43], and some of these factors may condition the response to fibrinolytic treatment. (3) Twenty percent of patients with lacunar syndrome do not develop lacunar infarction, and 5 to 30% of lacunar infarcts are the result of cerebral embolisms [44]. (4) The existence of penumbra in small vessel disease is questionable [45]. (5) The immediate and remote mechanisms of action of rt-PA may be different; due to the integrity of the blood-brain barrier, the rt-PA does not overflow and may have a neuroprotective effect or may condition a progression of white matter lesion, but not neuronal toxicity [46]. In the 21 patients with lacunar infarcts treated with rt-PA and excluded from our analysis, only 2 (9.5%) worsened in the first 3 months (data not shown), revealing the existence of a probably different rt-PA toxicity mechanism.

Our study has limitations, which, while not calling into question the validity of the results, imply the need for further prospective studies. We do not have the data on the dose of rt-PA given or the topography of the lesion (it is possible that neurotoxicity affects cortical and subcortical infarcts differently). Also, the best control group is not patients who do not receive fibrinolytic treatment but those who underwent thrombectomy without rt-PA, but the number of patients that meet this characteristic in our series was too small.

## Summary

In summary, administration of rt-PA intravenously or intraarterially may be associated with a higher risk of functional deterioration in the first 3 months if reperfusion within the first 24 h does not happen. We thus postulate that patients treated with rt-PA without an effective immediate reperfusion should be treated with appropriate drugs to neutralize possible adverse effects of rt-PA, including its ability to promote NMDA receptors and subsequent neurotoxicity.
